# Ingestion of *Lactobacillus intestinalis* and *Lactobacillus reuteri* causes depression- and anhedonia-like phenotypes in antibiotic-treated mice via the vagus nerve

**DOI:** 10.1186/s12974-020-01916-z

**Published:** 2020-08-15

**Authors:** Siming Wang, Tamaki Ishima, Jiancheng Zhang, Youge Qu, Lijia Chang, Yaoyu Pu, Yuko Fujita, Yunfei Tan, Xingming Wang, Kenji Hashimoto

**Affiliations:** Division of Clinical Neuroscience, Chiba University Center for Forensic Mental Health, 1-8-1 Inohana, Chiba, 260-8670 Japan

**Keywords:** Anhedonia, Antibiotic, Depression, Gut microbiota, Vagus nerve

## Abstract

**Background:**

The brain–gut–microbiota axis plays a role in the pathogenesis of stress-related disorders such as depression. In this study, we examined the effects of fecal microbiota transplantation (FMT) in mice with antibiotic-treated microbiota depletion.

**Methods:**

The fecal microbiota was obtained from mice subjected to chronic social defeat stress (CSDS) and control (no CSDS) mice. FMT from these two groups was performed to antibiotic-treated mice. 16S rRNA analysis was performed to examine the composition of gut microbiota. Furthermore, the effects of subdiaphragmatic vagotomy in depression-like phenotypes after ingestion of microbes were examined.

**Results:**

The ingestion of fecal microbiota from CSDS-susceptible mice resulted in an anhedonia-like phenotype, higher plasma levels of interleukin-6 (IL-6), and decreased expression of synaptic proteins in the prefrontal cortex (PFC) in antibiotic-treated mice but not in water-treated mice. 16S rRNA analysis suggested that two microbes (*Lactobacillus intestinalis* and *Lactobacillus reuteri*) may be responsible for the anhedonia-like phenotype in antibiotic-treated mice after FMT. Ingestion of these two microbes for 14 days led to depression- and anhedonia-like phenotypes, higher plasma IL-6 levels, and decreased expression of synaptic proteins in the PFC of antibiotic-treated mice. Interestingly, subdiaphragmatic vagotomy significantly blocked the development of behavioral abnormalities, elevation of plasma IL-6 levels, and downregulation of synaptic proteins in the PFC after ingestion of these two microbes.

**Conclusions:**

These findings suggest that microbiota depletion using an antibiotic cocktail is essential for the development of FMT-induced behavioral changes and that the vagus nerve plays a key role in behavioral abnormalities in antibiotic-treated mice after the ingestion of *L. intestinalis* and *L. reuteri*. Therefore, it is likely that the brain–gut–microbiota axis participates in the pathogenesis of depression via the vagus nerve.

## Background

The brain–gut–microbiota axis plays a fundamental role in host physiology, homeostasis, development, and metabolism [1–6]. Accumulating evidence has implicated an abnormal microbiota composition in the host gastrointestinal tract in the pathogenesis of stress-related disorders such as depression [7–15], and this abnormality could affect the antidepressant-like effects of certain compounds [16–21].

Instead of germ-free mice, antibiotic cocktail-induced microbiome depletion has been used to investigate the role of the gastrointestinal microbiota in pathological conditions such as Parkinson’s disease and depression [13, 15, 22–25]. Recently, we reported that microbiome depletion via antibiotic treatment contributed to resilience to anhedonia in mice subjected to chronic social defeat stress (CSDS) [15], suggesting that the brain–gut–microbiota axis plays a role in resilience versus susceptibility to CSDS. Furthermore, we reported that the transplantation of fecal microbiota from rats with an anhedonia-like phenotype aggravated depression- and anhedonia-like phenotypes in mice treated with an antibiotic cocktail [13]. Interestingly, the transplantation of fecal microbes from mice with depression into germ-free mice resulted in depression-like behaviors compared with the effects of the transplantation of fecal microbes obtained from control animals [9]. Collectively, it appears that the brain–gut–microbiota axis plays a key role in depression- and anhedonia-like phenotypes in rodents. However, the precise mechanisms underlying fecal microbiota transplantation (FMT)-induced behavioral abnormalities in rodents treated with an antibiotic cocktail remain unknown.

This study thus aimed to investigate the role of the brain–gut–microbiota axis in depression- and anhedonia-like phenotypes in mice. First, we examined whether transplantation of the fecal microbiota from CSDS-susceptible mice could induce an anhedonia-like phenotype in mice treated with an antibiotic cocktail. Using 16S rRNA analysis, we analyzed the composition of the gastrointestinal microbiota in fecal samples from these mice. We identified two microbes (*Lactobacillus intestinalis* and *Lactobacillus reuteri*) potentially responsible for the anhedonia-like phenotype in recipient mice. Second, we examined whether ingestion of these two microbes for 14 days produced depression- and anhedonia-like phenotypes in mice treated with an antibiotic cocktail. Furthermore, we measured the plasma levels of the inflammatory cytokine interleukin-6 (IL-6) and synaptic proteins (i.e., α-amino-3-hydroxy-5-methyl-4-isoxazolepropionic acid receptor A1 [GluA1] and postsynaptic density 95 [PSD-95]) in the prefrontal cortex (PFC) since the expression of these synaptic proteins was decreased in the PFC from rodents with depression-like phenotypes [26, 27]. Finally, we investigated whether subdiaphragmatic vagotomy (SDV) affected depression- and anhedonia-like phenotypes in mice treated with an antibiotic cocktail after the ingestion of these two microbes because the microbiota and brain are known to communicate through the vagus nerve [28–32].

## Materials and methods

### Animals

Male adult C57BL/6 mice (*n* = 120, 8 weeks old, body weight = 20–25 g, Japan SLC, Inc., Hamamatsu, Japan) and male adult CD1 (ICR) mice (*n* = 20, 13–15 weeks old, body weight > 40 g, Japan SLC, Inc.) were used. Animals were housed under controlled temperatures and 12-h/12-h light/dark cycles (lights on between 07:00 and 19:00 h) with ad libitum access to food (CE-2; CLEA Japan, Inc., Tokyo, Japan) and water. The protocol was approved by the Chiba University Institutional Animal Care and Use Committee (permission number: 30-399 and 1-456). This study was conducted in strict accordance with the recommendations in the Guide for the Care and Use of Laboratory Animals of the US National Institutes of Health. Animals were deeply anesthetized with isoflurane before being sacrificed via cervical dislocation. All efforts were made to minimize suffering. Transplantation of fecal samples and bacteria was performed from 16:00 to 17:00, and the 1% sucrose preference test (SPT) was performed from 17:00 to 18:00.

### CSDS

The CSDS procedure was performed as previously reported [15, 17–19, 33–37]. C57BL/6 mice were exposed to a different CD1 aggressor mouse for 10 min per day for 10 consecutive days (days 1–10). When the social defeat session ended, the resident CD1 mouse and intruder mouse were housed on opposite sides of the cage, separated by a perforated Plexiglass divider to allow visual, olfactory, and auditory contact for the remainder of the 24-h period. At 24 h after the last session, all mice were housed individually. On day 11, a social interaction test was performed to identify subgroups of mice that were susceptible and unsusceptible to CSDS. This was accomplished by placing mice in an interaction test box (42 × 42 cm^2^) with an empty wire-mesh cage (10 × 4.5 cm^2^) located at one end. The movement of the mice was tracked for 2.5 min, followed by 2.5 min in the presence of an unfamiliar aggressor confined in the wire-mesh cage. The duration of the subject’s presence in the “interaction zone” (defined as the 8-cm-wide area surrounding the wire-mesh cage) was recorded using a stopwatch. The interaction ratio was calculated as time spent in the interaction zone with an aggressor divided by the time spent in the interaction zone without an aggressor. An interaction ratio of 1 was set as the cutoff. Mice with scores < 1 were defined as “susceptible” to social defeat stress, and those with scores ≥ 1 were defined as “resilient.” Fresh fecal samples were collected from CSDS-susceptible mice and control (no CSDS) mice on days 12–14 in sterilized screw cap microtubes immediately after defecation and stored at − 80 °C until FMT. The fecal samples were collected from each mouse around 9:00–10:00 on each day to avoid circadian effects on the microbiome. In total, about 30 collected tubes containing approximately 0.5 g of feces per each tube were used for FMT. Before FMT, fecal samples were removed from the freezer every morning, and they were allowed to thaw for 10–15 min at room temperature. Then, drinking water (10 mL/g feces) was added to the tube including the fecal samples. The drinking water including fecal samples (0.2 mL/mouse) was given to the antibiotic-treated mice using gastric gavage for consecutive 14 days.

### Antibiotic cocktail treatment, FMT, and behavioral tests

Based on previous reports [13, 15, 24, 25], broad-spectrum antibiotics (ampicillin 1 g/L, neomycin sulfate 1 g/L, metronidazole 1 g/L, Sigma-Aldrich Co. Ltd, St. Louis, MO, USA) dissolved in drinking water were given ad libitum to male C57BL/6 mice for 14 consecutive days (days 1–14). The drinking solution was renewed every 2 days.

**Fig. 1:**
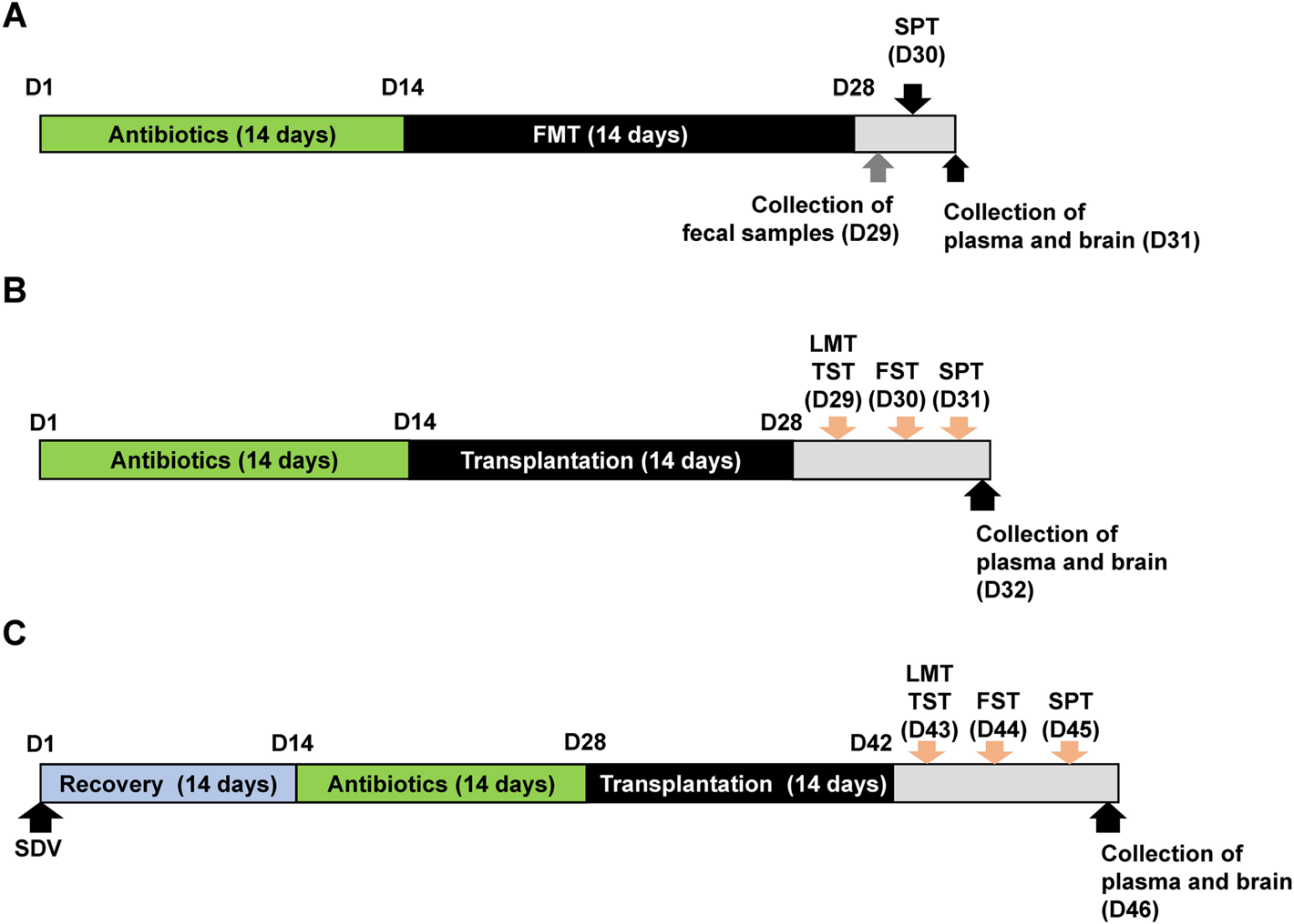
The schedule of the experiments. **a** The schedule of treatment of antibiotic cocktail, fecal microbiota transplantation (FMT), collection of fecal samples, sucrose preference test (SPT), and collection of the plasma and brain. Antibiotic cocktail or water in drinking water was given to adult male mice for 14 days (day 1–day 14). Subsequently, FMT from CSDS-susceptible mice or control (no CSDS) mice was performed for 14 days (day 15–day 28). On day 29, fecal samples were collected. On day 30, 1% SPT was performed. On day 31, the plasma and brain (i.e., PFC) were collected. **b** The schedule of treatment of antibiotic cocktail, microbiota transplantation, collection of fecal samples, behavioral tests, and collection of the plasma and brain. Antibiotic cocktail or water in drinking water was given to adult male mice for 14 days (day 1–day 14). Subsequently, two bacteria (*L. intestinalis* and *L. reuteri*) were administered orally for 14 days (day 15–day 28) using gastric gavage. On day 29, locomotion test (LMT) and tail suspension test (TST) were performed. Forced swimming test (FST) and 1% SPT were performed on days 30 and 31, respectively. On day 32, the plasma and brain were collected. **c** The schedule of subdiaphragmatic vagotomy (SDV), treatment of antibiotic cocktail, microbiota transplantation, behavioral tests, and collection of the plasma and brain. SDV or sham was performed in adult male mice, and mice were recovered 14 days after surgery (day 1–day 14). Subsequently, an antibiotic cocktail or water in drinking water was given to all mice for 14 days (day 15–day 28). Subsequently, transplantation of two microbiota was performed for 14 days (day 29–day 42). On day 43, LMT and TST were performed. FST and 1% SPT were performed on day 44 and day 45, respectively. On day 46, the plasma and brain were collected

Experiment 1 (Fig. 1a): Water alone or water containing the antibiotic cocktail was given to mice on days 1–14. Subsequently, mice were divided into four groups: water + FMT from control (no CSDS-susceptible) mice, water + FMT from CSDS-susceptible mice, antibiotic cocktail + FMT from control (no CSDS-susceptible) mice, and antibiotic cocktail + FMT from CSDS-susceptible mice. The fecal microbiota from CSDS-susceptible or control (no CSDS-susceptible) mice was administered orally from day 15 to day 28. On day 29, fecal samples were collected. The 1% SPT was performed on day 30. Blood samples were collected from the heart under isoflurane anesthesia and placed into a tube containing ethylenediamine-*N*,*N*,*N′*,*N*′-tetraacetic acid potassium salt dehydrate as an anticoagulant. Subsequently, blood samples were centrifuged (3000×*g*, 3 min) to prepare plasma samples. The plasma samples were stored at − 80 °C until assay. The brain region such as PFC was dissected from the brain on ice and stored at − 80 °C until use.

Experiment 2 (Fig. 1b): Mice were given drinking water alone or drinking water containing the antibiotic cocktail on days 1–14. Subsequently, mice were divided into three groups: water + water, antibiotic cocktail + water, and antibiotic cocktail + microbe (*L. intestinalis* and *L. reuteri*) groups. *L. intestinalis* (catalog number: JCM7548) and *L. reuteri* (catalog number: JCM1112) were purchased from RIKEN BioResource Research Center (Tsukuba, Ibaraki, Japan). Mice were orally administered water or water containing the microbes (approximately 1 × 10^8^ CFU/day) for 14 days (days 15–28) using gastric gavage. The locomotion test and tail suspension test (TST) were performed on day 29. The forced swimming test (FST) and 1% SPT were performed on days 30 and 31, respectively. On day 32, plasma samples and PFC samples were collected as described above and stored at − 80 °C until use.

Experiment 3 (Fig. 1c): Sham surgery or subdiaphragmatic vagotomy (SDV) was performed under anesthesia with 5% isoflurane. Mice were put under a microscope (Leica LEICA S9E, Germany), and hair was removed from the abdomen [38]. The esophagus of each mouse was exposed to the full view. The ventral and dorsal vagus nerves of the esophagus were severed. After the muscle and skin were sutured, mice were kept in clean cages until complete recovery from anesthesia. Then, mice were housed in cages for 14 days (days 1–14). The antibiotic cocktail was given to all mice in drinking water for 14 days (from day 15 to day 28). Subsequently, mice were divided into four groups: sham + water, SDV + water, SDV + microbe (*L. intestinalis* and *L. reuteri*), and sham + microbe (*L. intestinalis* and *L. reuteri*) groups. Water alone or water containing the microbes (approximately 1 × 10^8^ CFU/day) was administered orally for 14 days (day 29 to day 42) using gastric gavage. The locomotion test and TST were performed on day 43. The FST and 1% SPT were performed on days 44 and 45, respectively. On day 46, plasma samples and PFC tissues were collected and stored at − 80 °C until use.

#### Behavioral tests

Behavioral tests, including the locomotion test, TST, FST, and 1% SPT, were performed as previously reported [15, 17–19, 33–37].

Locomotion: Locomotor activity was measured using a SCANETMV-40 animal movement analysis system (MELQUEST Co., Ltd., Toyama, Japan). The animals were placed in experimental cages (560 × 560 × 330 mm^3^). Cumulative exercise was recorded for 60 min. The cages were cleaned after each testing session.

TST: A small piece of adhesive tape was placed approximately 2 cm from the tip of the tail of each mouse. A single hole was punched in the tape, and mice were hung individually on a hook. The immobility time was recorded for 10 min. Mice were considered immobile only when they hung passively and completely motionless.

FST: The FST was conducted using an automated forced-swim apparatus (SCANET MV-40; MELQUEST Co., Ltd.). Mice were placed individually in a cylinder (23 × 31 cm^2^) containing 15 cm of water maintained at a temperature of 23 ± 1 °C. The immobility time was calculated using the activity time as total time − active time by the apparatus analysis software. The immobility time of each mouse was recorded for a period of 6 min.

SPT: Mice were exposed to water and 1% sucrose solution for 48 h, followed by 4 h of water and food deprivation and a 1-h exposure to two identical bottles (water and 1% sucrose solution). The bottles containing water and sucrose were weighed before and at the end of this period. The sucrose preference was calculated as a percentage of sucrose solution consumption to the total liquid consumption.

### Measurement of the inflammatory cytokine IL-6

Plasma levels of IL-6 were measured because we previously identified an increase in blood IL-6 levels in the CSDS model [15, 18]. Plasma IL-6 levels were measured using an ELISA kit (cat#: 88-7064-22 Invitrogen, Carlsbad, CA, USA) according to the manufacturer’s instructions.

### Western blot analysis

PFC tissues were homogenized in the Laemmli lysis buffer. Aliquots (60 μg) of protein were measured using a DC protein assay kit (Bio-Rad, Hercules, CA, USA); incubated for 5 min at 95 °C with a quarter volume of 125 mM Tris-HCl, pH 6.8, 20% glycerol, 0.1% bromophenol blue, 10% β-mercaptoethanol, and 4% sodium dodecyl sulfate; and subjected to sodium dodecyl sulfate-polyacrylamide gel electrophoresis using mini-gels (catalog #: 4568126, Mini-PROTEAN TGX™ Stain-Free Gel; Bio-Rad). Proteins were transferred onto polyvinylidene difluoride membranes using a Trans-Blot Mini Cell (Bio-Rad). For immunodetection, the blots were blocked with 2% bovine serum albumin in TBS + 0.1% Tween-20 (TBST) for 1 h at room temperature and incubated with a primary antibody against GluA1 (catalog number: ab31232, 1 μg/mL, Abcam, Cambridge, MA, USA) and β-actin (catalog number: A5441 1:10,000; Sigma-Aldrich) overnight at 4 °C. The next day, blots were washed three times in TBST and incubated with horseradish peroxidase-conjugated anti-rabbit antibody (catalog number: NA934, GE Healthcare) and anti-mouse antibody (catalog number: NA931, GE Healthcare) for 1 h at room temperature. After three final washes with TBST, bands were detected using enhanced chemiluminescence plus a Western Blotting Detection system (GE Healthcare Bioscience). The blots then were incubated in stripping buffer (2% sodium dodecyl sulfate, 100 mM β-mercaptoethanol, and 62.5 mM Tris-HCl, pH 6.8) for 30 min at 60 °C and then washed three times with TBST. The stripped blots were kept in the blocking solution for 1 h and incubated with primary antibody directed against PSD-95 (catalog number: 51-6900, 1 μg/mL, Invitrogen). Images were captured using a ChemiDoc™ Touch Imaging System (170-01401; Bio-Rad Laboratories, Hercules, CA), and immunoreactive bands were quantified.

### 16S rRNA analysis and short-chain fatty acids

16S rRNA analysis of fecal samples was performed by MyMetagenome Co., Ltd. (Tokyo, Japan) as previously reported [15]. Measurement of short-chain fatty acids (i.e., acetic acid, propionic acid, butyric acid, lactic acid, and succinic acid) in fecal samples was performed at TechnoSuruga Laboratory, Co., Ltd. (Shizuoka, Japan) as previously reported [14, 15].

### Statistical analysis

The data are presented as the mean ± standard error of the mean (S.E.M.). Analysis was performed using PASW Statistics 20 (formerly SPSS statistics; SPSS, Tokyo, Japan). Comparisons between groups were performed via two-way analysis of variance (ANOVA) or one-way ANOVA, followed by post hoc Fisher’s least significant difference (LSD) test. The data for body weight were analyzed using repeated-measures two-way ANOVA, followed by post hoc Fisher LSD test. *P* < 0.05 was considered statistically significant.

## Results

### FMT from CSDS-susceptible mice and control (no CSDS) mice

**Fig. 2:**
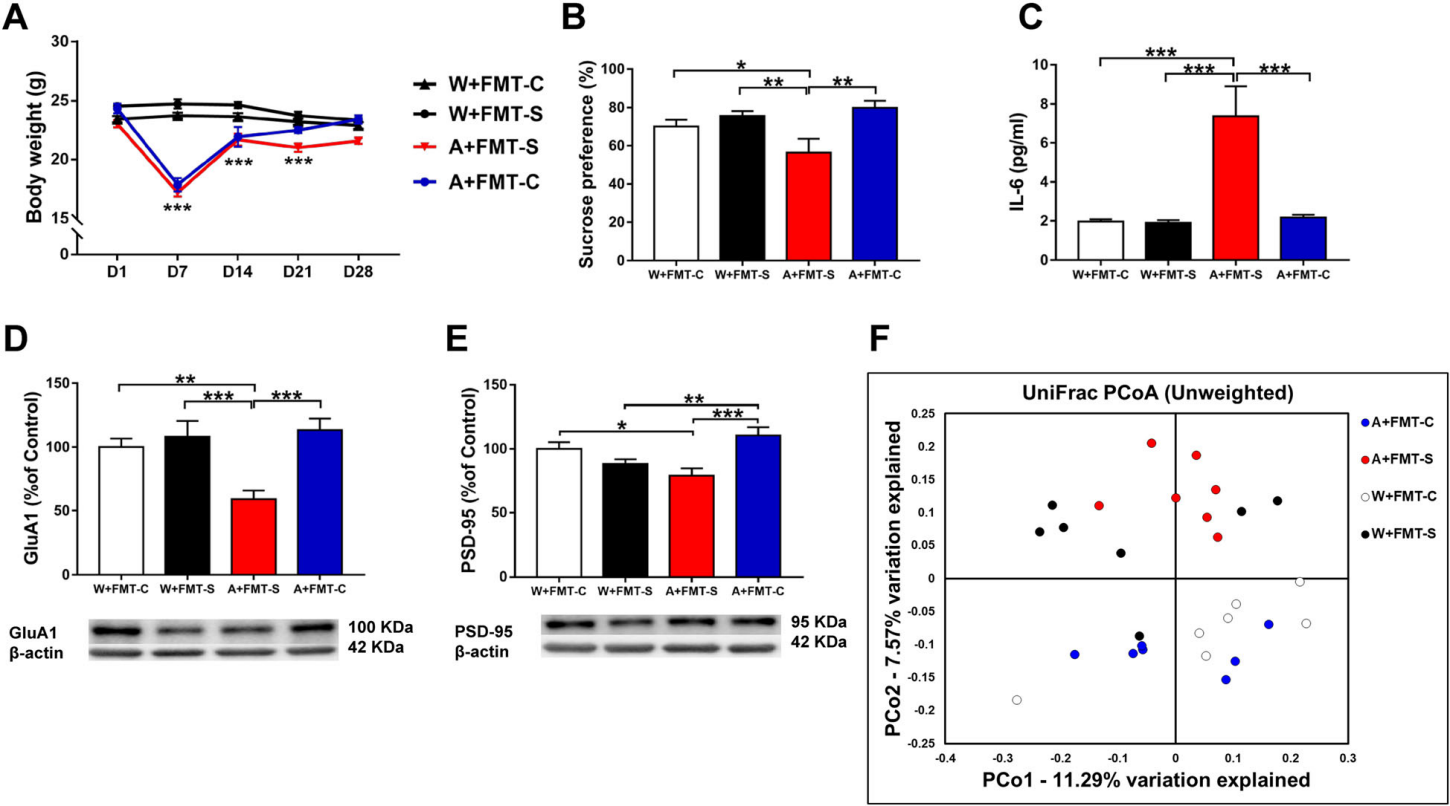
Effects of FMT in antibiotic-treated and water-treated mice. **a** Body weight (repeated measure two-way ANOVA, antibiotic: *F*_1,24_ = 38.007, *P* < 0.001; FMT: *F*_1,24_ = 0.667, *P* = 0.422; interaction: *F*_1,24_ = 2.282, *P* = 0.144). **b** SPT (two-way ANOVA, antibiotic: *F*_1,24_ = 1.021, *P* = 0.322; FMT: *F*_1,24_ = 3.722, *P* = 0.066; interaction: *F*_1,24_ = 9.757, *P* = 0.005). **c** Plasma IL-6 (two-way ANOVA, antibiotic: *F*_1,24_ = 13.300, *P* = 0.001; FMT: *F*_1,24_ = 10.919, *P* = 0.003; interaction: *F*_1,24_ = 11.393, *P* = 0.003). **d** GluA1 (two-way ANOVA, antibiotic: *F*_1,24_ = 3.833, *P* = 0.062; FMT: *F*_1,24_ = 6.437, *P* = 0.018; interaction: *F*_1,24_ = 11.657, *P* = 0.002). **e** PSD-95 (two-way ANOVA, antibiotics: *F*_1,24_ = 0.014, *P* = 0.908; FMT: *F*_1,24_ = 15.604, *P* = 0.001; interaction: *F*_1,24_ = 3.284, *P* = 0.082). **f** PCoA analysis of gut microbiota data. Data are shown as mean ± S.E.M. (*n* = 7). *P < 0.05, **P < 0.01, ***P < 0.001. FMT fecal microbiota transplantation, NS not significant, W + FMT-C water + FMT from control (no CSDS) mice, W + FMT-S water + FMT from CSDS-susceptible mice, A + FMT-S antibiotic + FMT from CSDS-susceptible mice, A + FMT-C antibiotic + FMT from control (no CSDS) mice

The first experiment examined the effects of FMT from CSDS-susceptible mice (Fig. 1a). Treatment with an antibiotic cocktail significantly decreased the body weight of the mice (Fig. 2a). Meanwhile, FMT did not result in body weight changes in CSDS-susceptible mice (Fig. 2a). In the 1% SPT, FMT from CSDS-susceptible mice caused significant reductions in sucrose preference in antibiotic-treated mice but not in water-treated mice (Fig. 2b). In contrast, FMT from control (no CSDS) mice did not cause significant reductions in sucrose preference in antibiotic-treated and water-treated mice (Fig. 2b). Among CSDS-susceptible mice, FMT resulted in significant increases in plasma IL-6 levels in the antibiotic-treated group but not in the water-treated group (Fig. 2c). Furthermore, FMT from CSDS-susceptible mice significantly decreased PSD-95 and GluA1 expression in the PFC in the antibiotic-treated group but not in the water-treated group (Fig. 2d, e).

**Fig. 3:**
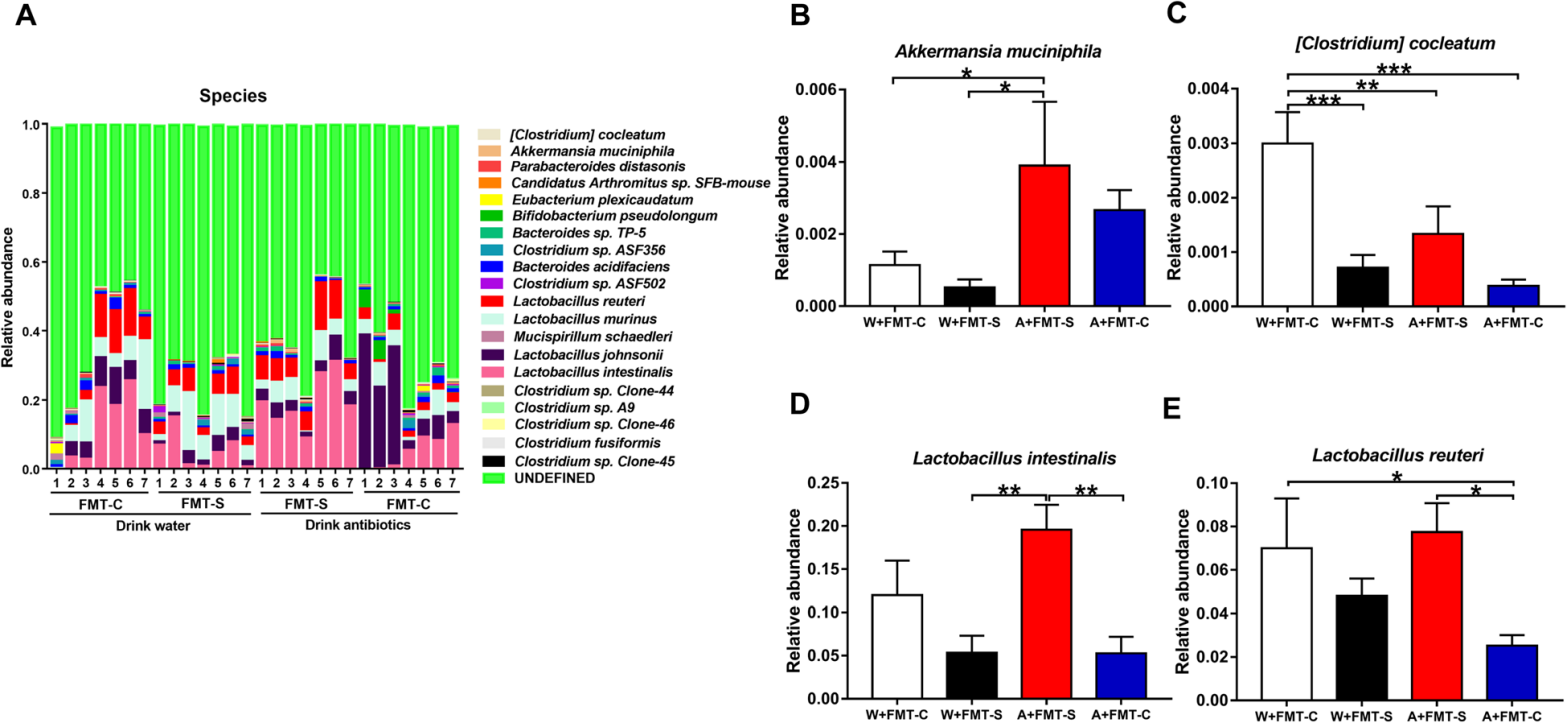
Altered composition in the gut microbiota at the species level. **a** The relative abundances of species in fecal samples of the four groups 24 h after the final FMT. **b** *Akkermansia muciniphila* (two-way ANOVA, antibiotic: *F*_1,24_ = 6.721, *P* = 0.016; FMT: *F*_1,24_ = 0.107, *P* = 0.746; interaction: *F*_1,24_ = 0.963, *P* = 0.336). **c** *[Clostridium] cocleatum* (two-way ANOVA, antibiotic: *F*_1,24_ = 6.153, *P* = 0.021; FMT: *F*_1,24_ = 2.733, *P* = 0.111; interaction: *F*_1,24_ = 16.133, *P* = 0.001). **d** *Lactobacillus intestinalis* (two-way ANOVA, antibiotic: *F*_1,24_ = 1.753, *P* = 0.198; FMT: *F*_1,24_ = 1.830, *P* = 0.189; interaction: *F*_1,24_ = 13.700, *P* = 0.001). **e** *Lactobacillus reuteri* (two-way ANOVA, antibiotic: *F*_1,24_ = 0.298, *P* = 0.590; FMT: *F*_1,24_ = 1.154, *P* = 0.293; interaction: *F*_1,24_ = 6.966, *P* = 0.014). Data are shown as mean ± S.E.M. (*n* = 7). *P < 0.05, **P < 0.01, ***P < 0.001. FMT fecal microbiota transplantation, NS not significant, W + FMT-C water + FMT from control (no CSDS) mice, W + FMT-S water + FMT from CSDS-susceptible mice, A + FMT-S antibiotic + FMT from CSDS-susceptible mice, A + FMT-C antibiotic + FMT from control (no CSDS) mice

Analysis of 16S rRNA was used to identify differences in the composition of the gastrointestinal microbiota among the four groups. Unweighted UniFrac-based principal coordinate analysis (PCoA) revealed significant differences among the four groups (Fig. 2f). *Firmicutes* and *Bacteroidetes* were the most abundant phyla in all groups (Figure S1A). At the phylum level, *Verrucomicrobia* was present at significantly high levels in the antibiotic + FMT group than in the water-treated groups (Figure S1B). Four genera of bacteria (*Akkermansia*, *Alistipes*, *Candidatus Arthromitus*, and *Parabacteroides*) were present at different levels among the four groups (Figure S2). At the species level, five bacteria (*Clostridium cocleatum*, *Akkermansia muciniphila*, *Lactobacillus intestinalis*, *Candidatus Arthromitus* sp. SFB-mouse, and *Lactobacillus reuteri*) were present at different levels among the four groups (Fig. 3). There were no differences in short-chain fatty acid levels (i.e., acetic acid, butyric acid, lactic acid, succinic acid, propionic acid) among the four groups (Figure S3). However, there was a positive correlation (*r* = 0.397, *P* = 0.045) between the presence of *L. intestinalis* and succinic acid levels in all groups (Figure S3).

Overall, FMT from CSDS-susceptible mice induced an anhedonia-like phenotype, inflammation, and downregulation of synaptic proteins in the PFC in the antibiotic-treated group. Among these bacteria, *L. intestinalis* and *L. reuteri* may be involved in the anhedonia-like phenotype, upregulation of IL-6, and downregulation of synaptic proteins in the PFC after FMT from CSDS-susceptible mice.

### Oral administration of *L. intestinalis* and *L. reuteri* in antibiotic-treated mice

**Fig. 4:**
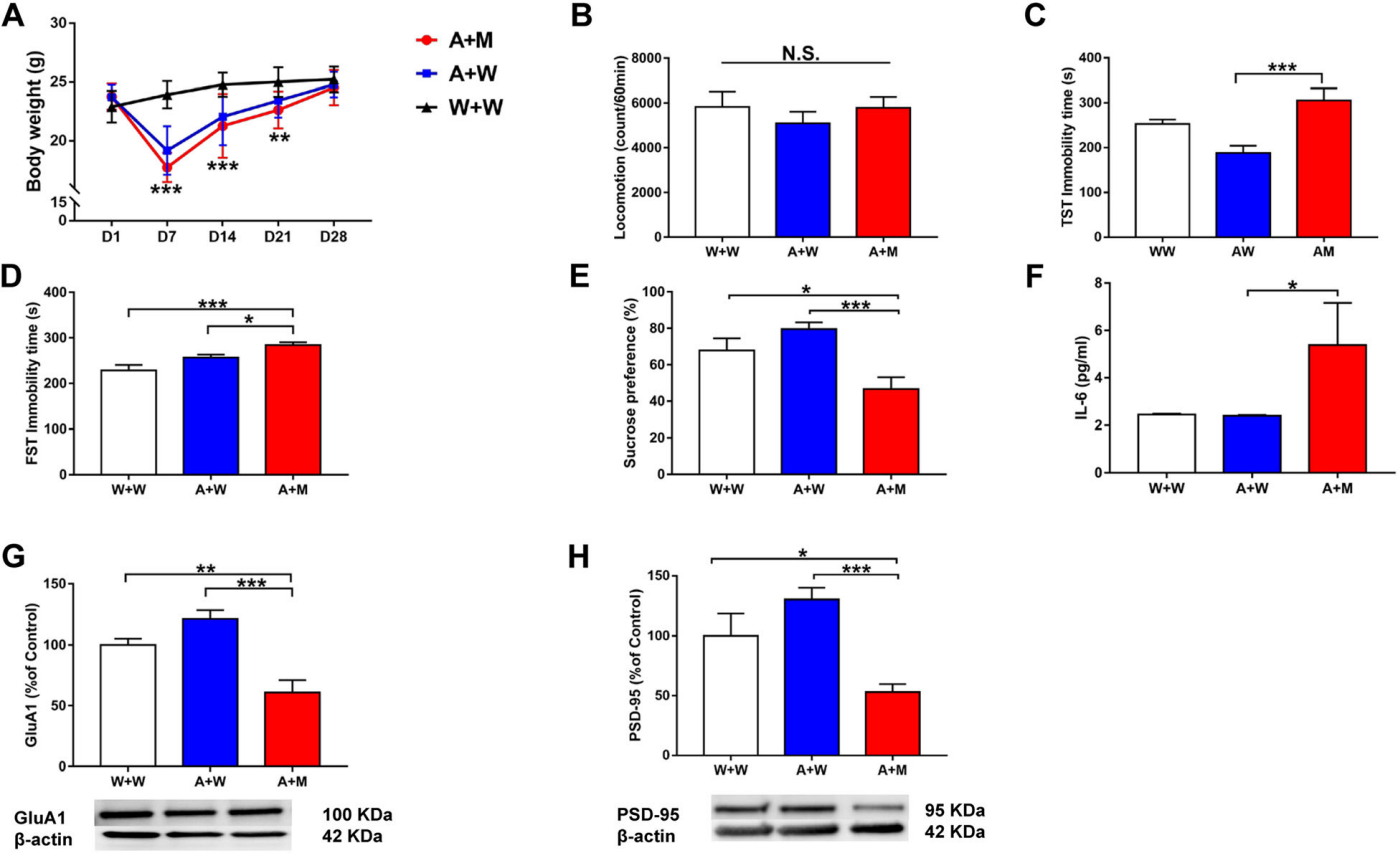
Effects of *L. intestinalis* and *L. reuteri* in the antibiotic-treated mice. **a** Body weight (repeated measure two-way ANOVA, antibiotic: *F*_1,27_ = 9.281, *P* = 0.005; transplantation: *F*_1,27_ = 1.284, *P* = 0.267). **b** Locomotion (LMT) (one-way ANOVA, *F*_2,27_ = 0.533, *P* = 0.593). **c** TST (one-way ANOVA, *F*_2,27_ = 9.695, *P* = 0.001). **d** FST (one-way ANOVA, *F*_2,27_ = 10.749, *P* < 0.001). **e** SPT (one-way ANOVA, *F*_2,27_ = 8.214, *P* = 0.002). **f** Plasma IL-6 (one-way ANOVA, *F*_2,27_ = 2.775, *P* = 0.080). **g** GluA1 (one-way ANOVA, *F*_2,27_ = 15.481, *P* < 0.001). **h** PSD-95 (one-way ANOVA, *F*_2,27_ = 9.185, *P* = 0.001). Data are shown as mean ± S.E.M. (*n* = 10). *P < 0.05, **P < 0.01, ***P < 0.001. NS not significant, W + W water + water-treated mice, A + W antibiotic + water-treated mice, A + M antibiotic + microbiota transplantation

Next, we investigated whether two microbes (*L. intestinalis* and *L. reuteri*) could induce depression-like and anhedonia-like phenotypes in mice treated with an antibiotic cocktail (Fig. 1b). Treatment with an antibiotic cocktail significantly decreased the body weight of mice (Fig. 4a). Ingestion of the two microbes did not alter body weight in antibiotic-treated mice (Fig. 4a). There were no changes in locomotion among the three groups (Fig. 4b). The immobility times in the antibiotic + microbe group as determined using the TST and FST were significantly higher than those in the control and antibiotic + water groups (Fig. 4c, d). Furthermore, the sucrose preference in the antibiotic + microbe group was significantly lower than that in the control and antibiotic + water groups (Fig. 4e). Moreover, the expression of synaptic proteins (i.e., GluA1 and PSD-95) in the PFC was significantly lower in the antibiotic + microbe group than in the control and antibiotic + water groups (Fig. 4g, h). In addition, the blood levels of IL-6 were significantly higher in the antibiotic + microbe group than in the control and antibiotic + water groups (Fig. 4f).

**Fig. 5:**
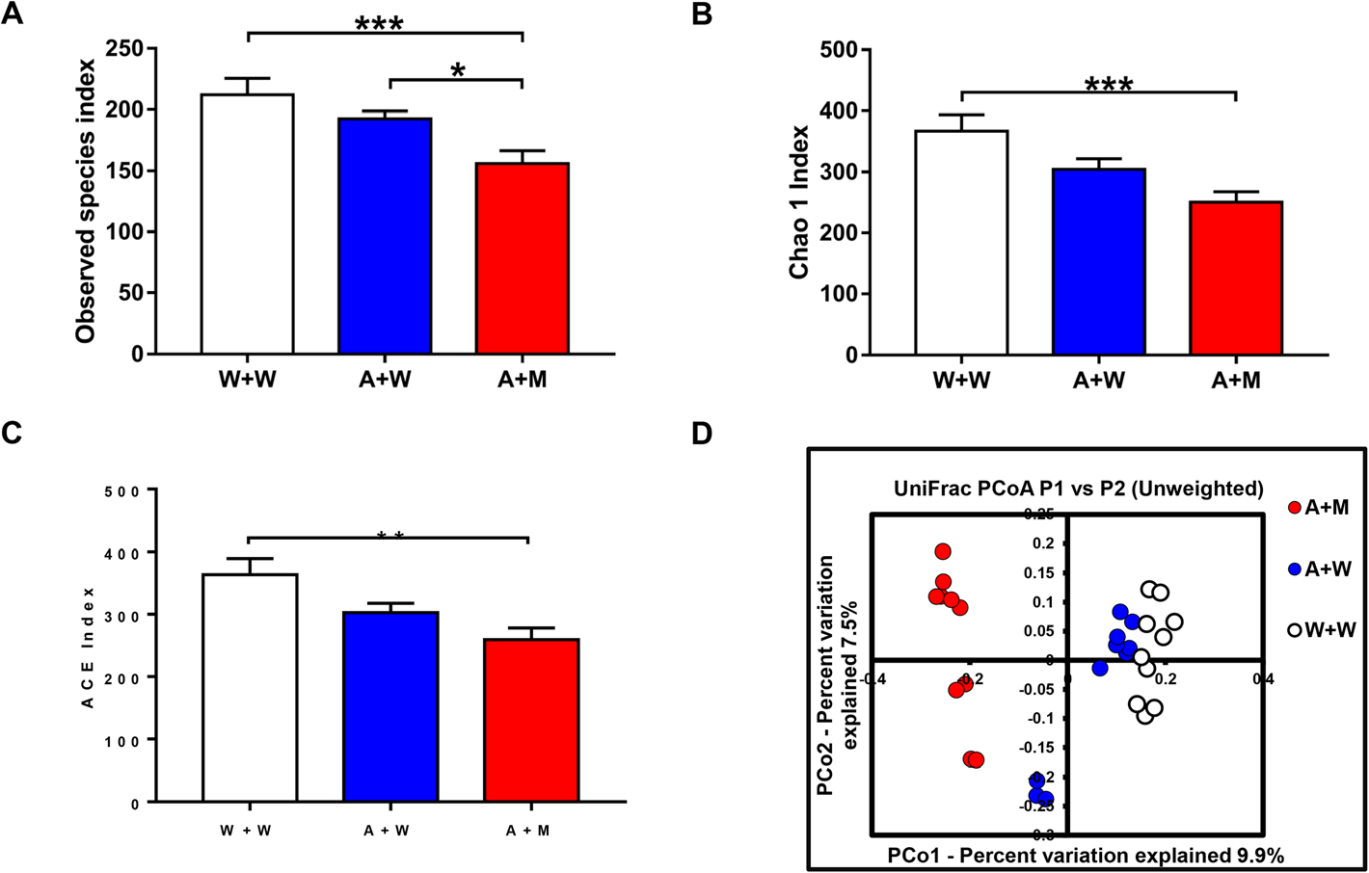
Effects of transplantation of *L. intestinalis* and *L. reuteri* in the antibiotic-treated mice. **a** Observed species index (one-way ANOVA, *F*_2,27_ = 7.381, *P* = 0.003). **b** Chao 1 index (one-way ANOVA, *F*_2,27_ = 7.811, *P* = 0.002). **c** ACE index (one-way ANOVA, *F*_2,27_ = 6.642, *P* = 0.005). **d** PCoA analysis of gut bacteria data. Data are shown as mean ± S.E.M. (*n* = 10). *P < 0.05, ***P < 0.001. NS not significant

Next, we performed 16S rRNA analysis of fecal samples after transplantation of the two microbes. There were significant differences in the examined species indices among the three groups (Fig. 5a). The Chao1 and abundance-based coverage estimator (ACE) indices are used to evaluate the α-diversity of the gastrointestinal microbiota. These indices were significantly different among the three groups (Fig. 5b, c). Interestingly, the transplantation of the two microbes decreased the Chao1 and ACE indices. In unweighted UniFrac-based PCoA, dots representing the antibiotic + microbe group were far from those representing the other two groups (Fig. 5d).

These data suggest that oral administration of these two bacteria for 14 days induced depression- and anhedonia-like phenotypes, inflammation, and synaptic protein downregulation in the PFC in mice treated with an antibiotic cocktail.

### Effects of SDV on behavioral abnormalities, inflammation, and decreased expression of synaptic proteins in the PFC of antibiotic-treated mice after ingestion of *L*. *intestinalis* and *L*. *reuteri*

**Fig. 6:**
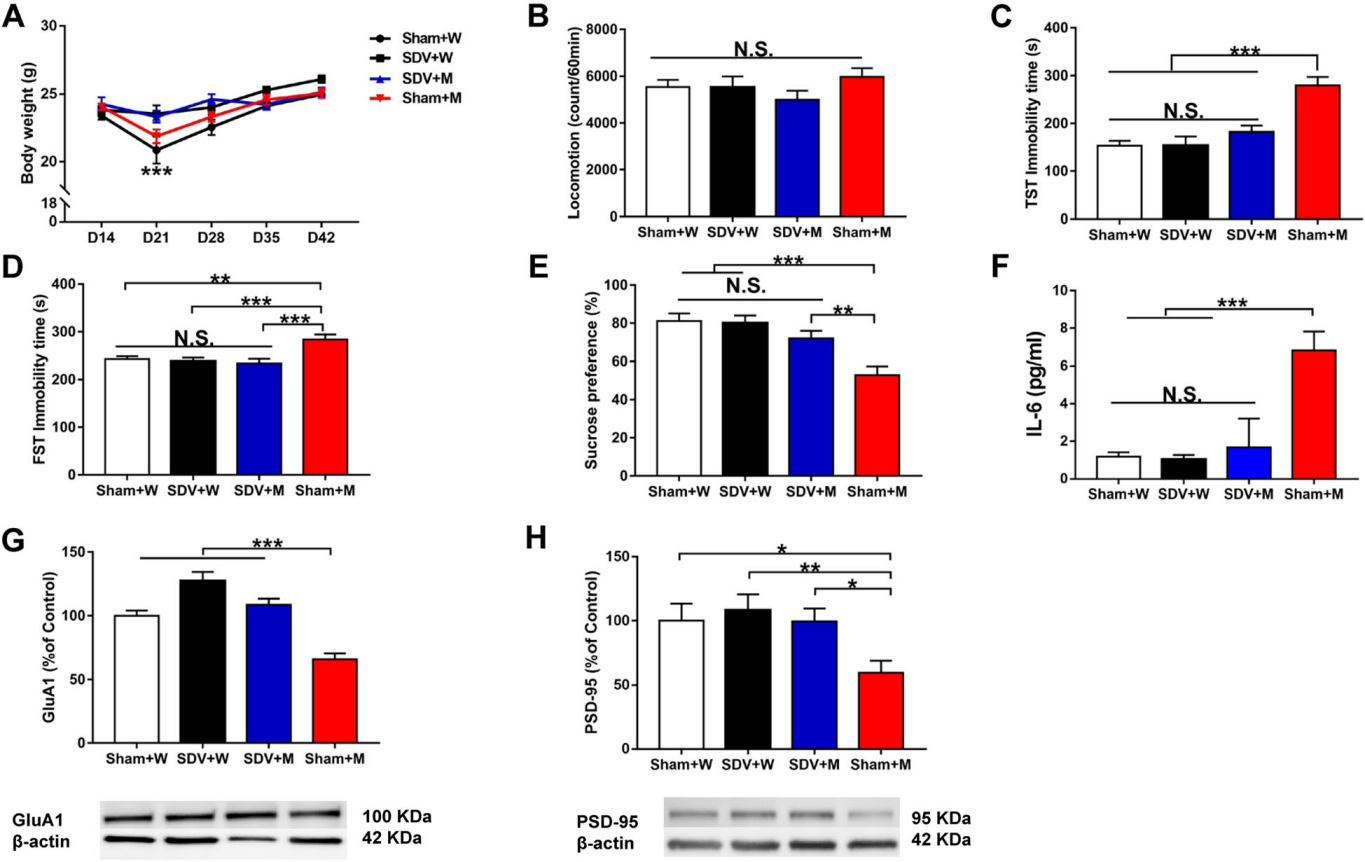
Effects of SDV on behavioral abnormalities after transplantation of *L. intestinalis* and *L. reuteri* in the antibiotic-treated mice. **a** Body weight (repeated measure two-way ANOVA, SDV: *F*_1,28_ = 8.910, *P* = 0.006; microbiota transplantation: *F*_1,28_ = 0.317, *P* = 0.578; interaction: *F*_1,28_ = 1.713, *P* = 0.201). **b** Locomotion (two-way ANOVA, SDV: *F*_1,28_ = 1.716, *P* = 0.201; microbiota transplantation: *F*_1,28_ = 0.015, *P* = 0.905; interaction: *F*_1,28_ = 1.649, *P* = 0.210). **c** TST (two-way ANOVA, SDV: *F*_1,28_ = 9.916, *P* = 0.004; microbiota transplantation: *F*_1,28_ = 25.222, *P* < 0.001; interaction: *F*_1,28_ = 10.069, *P* = 0.004). **d** FST (two-way ANOVA, SDV: *F*_1,28_ = 10.181, *P* = 0.003; microbiota transplantation: *F*_1,28_ = 4.893, *P* = 0.035; interaction: *F*_1,28_ = 6.957, *P* = 0.013). **e** SPT (two-way ANOVA, SDV: *F*_1,28_ = 4.820, *P* = 0.037; microbiota transplantation: *F*_1,28_ = 19.414, *P* < 0.001; interaction: *F*_1,28_ = 5.943, *P* = 0.021). **f** Plasma IL-6 (two-way ANOVA, SDV: *F*_1,28_ = 64.671, *P* < 0.001; microbiota transplantation: *F*_1,28_ = 92.048, *P* < 0.001; interaction: *F*_1,28_ = 57.921, *P* < 0.001). **g** GluA1 (two-way ANOVA, SDV: *F*_1,28_ = 43.231, *P* < 0.001; microbiota transplantation: *F*_1,28_ = 24.521, *P* < 0.001; interaction: *F*_1,28_ = 2.093, *P* = 0.159). **h** PSD-95 (two-way ANOVA, SDV: *F*_1,28_ = 4.376, *P* = 0.046; microbiota transplantation: *F*_1,28_ = 4.560, *P* = 0.042; interaction: *F*_1,28_ = 1.945, *P* = 0.174). Data are shown as mean ± S.E.M. (*n* = 8). *P < 0.05, **P < 0.01, ***P < 0.001. NS not significant, sham + W sham + water-treated mice, SDV + W SDV + water-treated mice, SDV + M SDV + microbiota transplantation, sham + M sham + microbiota transplantation

We investigated the effects of SDV on abnormal behaviors and inflammation in antibiotic-treated mice after the ingestion of *L. intestinalis* and *L. reuteri* (Fig. 1c). Body weight was not significantly different between before and 14 days after SDV (Fig. 6a). Treatment with an antibiotic cocktail significantly decreased body weight in the sham-treated groups (sham + water and sham + microbes) but not in SDV-treated groups (SDV + water and SDV + microbes) on day 21 (Fig. 6a). There were no changes in body weight from day 28 to day 42 (Fig. 6a). There were no changes in locomotion among the four groups (Fig. 6b). The immobility times in the SDV + microbe group as determined using the TST and FST were significantly lower than those in the sham + microbe group (Fig. 6c, d). Furthermore, the sucrose preference was significantly higher in the SDV + microbe group than in the sham + microbe group (Fig. 6e). Moreover, GluA1 and PSD-95 expression in the PFC was significantly higher in the SDV + microbe group than in the sham + microbe group (Fig. 6g, h). Conversely, IL-6 blood levels were significantly lower in the SDV + microbe group than in the sham + microbe group (Fig. 6f).

These data suggest that SDV blocked the development of depression- and anhedonia-like phenotypes, inflammation, and synaptic protein downregulation in the PFC in antibiotic-treated mice after the ingestion of *L. intestinalis* and *L. reuteri*.

## Discussion

The major findings of this study were as follows. First, FMT from CSDS-susceptible mice caused an anhedonia-like phenotype, inflammation, and synaptic protein downregulation in the PFC among those treated with antibiotics but not those treated with water. Thus, antibiotic-induced microbiota depletion in the host is essential for the development of FMT-induced behavioral and biochemical changes in recipient mice. 16S rRNA analysis suggested that among antibiotic-treated mice, *L. intestinalis* and *L. reuteri* counts were higher in the FMT group from CSDS-susceptible mice than in the FMT group from control mice, suggesting that these two bacteria may play a role in the anhedonia-like phenotype, inflammation, and reduction of synaptic protein expression in the PFC. Second, ingestion of *L. intestinalis* and *L. reuteri* for 14 days induced depression- and anhedonia-like phenotypes, inflammation, and synaptic protein downregulation in the PFC among antibiotic-treated mice. In unweighted UniFrac PCoA, dots representing the antibiotic + microbe group were distantly separated from dots representing the two water groups. Transplantation of the two microbes caused significant changes in the diversity and composition of the host gastrointestinal microbiota. Third, SDV significantly attenuated the depression- and anhedonia-like phenotypes, inflammation, and downregulation of synaptic proteins in the PFC in antibiotic-treated mice after the repeated ingestion of *L. intestinalis* and *L. reuteri*, suggesting a key role of the vagus nerve in the behavioral abnormalities and inflammation induced by oral administration of these two bacteria*.* Collectively, it is likely that *L. intestinalis* and *L. reuteri* produces depression-like and anhedonia-like phenotypes in antibiotic-treated mice through the brain–gut–microbiota axis via the vagus nerve.

In this study, we found that fecal microbes isolated from CSDS-susceptible mice did not induce anhedonia-like phenotypes, increased plasma IL-6 production, and reduced synaptic protein expression in the PFC in water-treated mice, whereas such changes were induced by these microbes in antibiotic-treated mice. Thus, antibiotic-induced microbiome depletion is necessary for the anhedonia-like phenotype and biochemical changes in recipient mice after FMT. Collectively, FMT in antibiotic-treated mice using “depression-related microbes” obtained from CSDS-susceptible mice resulted in anhedonia-like behaviors and reduced synaptic protein expression in the PFC through systemic inflammation. It is likely that the microbiota in the host gastrointestinal tract of water-treated mice could protect against the effects of FMT using “depression-related microbes” isolated from CSDS-susceptible mice. The mechanisms underlying anhedonia-like phenotypes in antibiotic-treated mice caused by FMT using depression-related microbes are currently unknown. Previously, we reported that CSDS failed to increase plasma IL-6 levels and the expression of synaptic proteins, such as GluA1 and PSD-95, in antibiotic-treated mice, although the same stress increased plasma IL-6 levels and reduced synaptic protein expression in water-treated mice [15]. This study suggests that the host gastrointestinal microbes are necessary for CSDS-induced increases in circulating cytokines and decreased expression of synaptic proteins in the PFC.

In this study, we found that *L. intestinalis* and *L. reuteri* counts were higher in the FMT group from CSDS-susceptible mice than in the FMT group from control (no CSDS) mice, suggesting that these two bacteria play a role in the anhedonia-like phenotype, inflammation, and reduced synaptic protein expression in the PFC. Ingestion of these two microbes for 14 days induced depression- and anhedonia-like phenotypes, increased blood IL-6 levels, and reduced synaptic protein expression in the PFC in antibiotic-treated mice. These data suggest that these two microbes induce depression- and anhedonia-like phenotypes and reduce synaptic protein expression in the PFC of antibiotic-treated mice through systemic inflammation even though these mice were not exposed to stress.

Treatment with a broad-spectrum antibiotic cocktail is known to cause a dramatic loss in the diversity and representation of specific taxa, increase the prevalence of antibiotic-resistant strains, and upregulate antibiotic resistance genes [39, 40]. Furthermore, Yang et al. [13] reported that FMT from anhedonia-susceptible rats into antibiotic-treated mice significantly exaggerated depression-like phenotypes including anhedonia and that FMT from resilient rats into antibiotic-treated mice significantly improved depression-like phenotype including anhedonia. Although the precise mechanisms underlying the abnormal composition of the gastrointestinal microbiota after treatment with an antibiotic cocktail are currently unknown, antibiotic-induced microbiome depletion is essential for behavioral abnormalities in recipients after FMT using microbes from CSDS-susceptible mice.

Previously, we reported that CSDS significantly increased the blood levels of IL-6 in water-treated mice but not in antibiotic-treated mice, suggesting that antibiotic-induced microbiota depletion has anti-inflammatory effects in mice [15]. Furthermore, CSDS significantly decreased the expression of synaptic proteins such as PSD-95 and GluA1 in the PFC in the water-treated group but not in the antibiotic-treated group. These findings suggest that antibiotic-induced microbiome depletion may contribute to stress resilience in mice after CSDS via the brain–gut–microbiome axis [15]. In this study, transplantation of two microbes caused depression- and anhedonia-like phenotypes in antibiotic-treated mice through systemic inflammation. These data suggest that these two microbes facilitate the development of behavioral abnormalities in antibiotic-treated mice, although the precise mechanisms remain unclear. Further detailed study is required to confirm the relationship between these two microbes and antibiotic-induced microbiota depletion in the host gastrointestinal tract.

The crosstalk between the brain and the gastrointestinal microbiota is predominately influenced through various routes including the vagus nerve, immune system, and enteric nervous system [28–32]. It has been demonstrated that the ingestion of *Lactobacillus rhamnosus* reduced stress-induced corticosterone levels and anxiety- and depression-related behaviors in mice and that these neurochemical and behavioral effects were not found in vagotomized mice, suggesting a role of the vagus nerve in the communication between the gastrointestinal microbiome and the brain [41]. The subdiaphragmatic vagus nerve serves as a major modulatory pathway between the brain and gut microbiota. Very recently, we demonstrated that lipopolysaccharide produces a depression-like phenotype and abnormal composition of gut microbiota via the subdiaphragmatic vagus nerve [38]. In this study, we found that the ingestion of *L. intestinalis* and *L. reuteri* did not induce depression- and anhedonia-like behaviors, increase plasma IL-6 levels, or reduce synaptic protein expression in the PFC in vagotomized mice. Taken all together, it is likely that the subdiaphragmatic vagus nerve plays a key role in behavioral abnormalities in rodents after the transplantation of these two microbes.

*L. reuteri* is a well-studied probiotic bacterium that can colonize a large number of mammals. It is well recognized that *L. reuteri* has several beneficial effects on anti-microbial activity, the host immune system, and microbial translocation [42]. It is reported that *L. reuteri* can rescue social dysfunction in a mouse model of autism in a vagus nerve-dependent manner [43]. Conversely, we found that ingestion of *L. reuteri* and *L. intestinalis* into antibiotic-treated mice caused depression-like behaviors via systemic inflammation. Thus, *L. reuteri* may have detrimental effects in the host gastrointestinal tract after antibiotic-induced microbiota depletion. Nonetheless, further study is needed to investigate the beneficial and detrimental effects of *L. reuteri* in mammals.

## Conclusions

The present study suggests that FMT using microbes from CSDS-susceptible mice induced depression- and anhedonia-like phenotypes in antibiotic-treated mice and that ingestion of *L. intestinalis* and *L. reuteri* caused behavioral and biochemical abnormalities in antibiotic-treated mice via the subdiaphragmatic vagus nerve. It is likely that the brain–gut–microbiota axis plays a role in the pathology of depression via the subdiaphragmatic vagus nerve.

## Supplementary information

**Additional file 1: Figure S1.** Altered composition in the gut microbiota at the phylum level. (A): The relative abundances of phylum in fecal samples of the four groups 24 hrs after the final FMT. (B): *Verrucomicrobi* (two-way ANOVA, antibiotic: F_1,24_ = 6.769, P = 0.016, FMT: F_1,24_ = 0.128, P = 0.724, interaction: F_1,24_ = 1.053, P = 0.315). Data are shown as mean ± S.E.M. (n = 7). *P < 0.05. FMT: fecal microbiota transplantation. NS: not significant. W + FMT-C: water + FMT from control (no CSDS) mice. W + FMT-S: water + FMT from CSDS susceptible mice. A + FMT-S: antibiotic + FMT from CSDS susceptible mice. A + FMT-C: antibiotic + FMT from control (no CSDS) mice. **Figure S2.** Altered composition in the gut microbiota at the genus level. (A): The relative abundances of genus in fecal samples of the four groups 24 hrs after the final FMT. (B): *Akkermansia* (two-way ANOVA, antibiotic: F_1,24_ = 6.721, P = 0.016, FMT: F_1,24_ =0.107, P = 0.746, interaction: F_1,24_ = 0.963, P = 0.336). (C): *Alistipes* (two-way ANOVA, antibiotic: F_1,24_ = 11.641, P = 0.002, FMT: F_1,24_ = 9.142, P = 0.006, interaction: F_1,24_ = 3.879, P = 0.061). (D): *Candidatus Arthromitus* (two-way ANOVA, antibiotic: F_1,24_ = 1.064, P =0.313, FMT: F_1,24_ = 5.899, P = 0.023, interaction: F_1,24_ = 1.356, P = 0.256). (E): *Parabacteroides* (two-way ANOVA, antibiotic: F_1,24_ = 0.665, P =0.423, FMT: F_1,24_ = 9.407, P = 0.005, interaction: F_1,24_ = 3.961, P = 0.058). Data are shown as mean ± S.E.M. (n = 7). *P< 0.05, **P < 0.01. FMT: fecal microbiota transplantation. NS: not significant. W + FMT-C: water + FMT from control (no CSDS) mice. W + FMT-S: water + FMT from CSDS susceptible mice. A + FMT-S: antibiotic + FMT from CSDS susceptible mice. A + FMT-C: antibiotic + FMT from control (no CSDS) mice. **Figure S3.** Levels of short-chain fatty acids in fecal samples and correlation with microbiota. (A): Acetic acid (two-way ANOVA, antibiotics: F_1,24_ = 0.170, P =0.684, FMT: F_1,24_ =1.028, P =0.321, interaction: F_1,24_ =0.170, P =0.683) among the four groups. (B): Butyric acid (two-way ANOVA, antibiotics: F_1,23_ = 0.831, P =0.372, FMT: F_1,23_ =0.497, P =0.488, interaction: F_1,23_=0.122, P =0.730) among the four groups. (C): Lactic acid (two-way ANOVA, antibiotics: F_1,23_ = 0.248, P =0.623, FMT: F_1,23_ =0.038, P =0.847, interaction: F_1,23_=0.782, P =0.386) among the four groups. (D): Succinic acid (two-way ANOVA, antibiotics: F_1,23_ = 0.511, P =0.482, FMT: F_1,23_ =0.970, P =0.355, interaction: F_1,23_=2.053, P =0.165) among the four groups. (E): Propionic acid (two-way ANOVA, antibiotics: F_1,24_ = 0.095, P =0.761, FMT: F_1,24_ =0.003, P =0.959, interaction: F_1,24_ =1.325, P =0.261) among the four groups. (F): There is a positive correlation (r = 0.397, P = 0.045) between succinic acid and *L. intestinalis* in fecal samples. The data are shown as mean ± S.E.M. (n = 7). FMT: fecal microbiota transplantation. NS: not significant.

## Data Availability

The data during the current study are available from the corresponding author upon reasonable request.

## Abbreviations

ACE: Abundance-based coverage estimator
ANOVA: Analysis of variance
CSDS: Chronic social defeat stress
FMT: Fecal microbiome transplantation
FST: Forced swimming test
GluA1: α-Amino-3-hydroxy-5-methyl-4-isoxazolepropionic acid receptor A1
IL-6: Interleukin-6
PCoA: Principal coordinate analysis
PFC: Prefrontal cortex
PSD-95: Postsynaptic density 95
SDV: Subdiaphragmatic vagotomy
SPT: Sucrose preference test
TBST: Tris buffer saline with 0.1% Tween-20
TST: Tail suspension test
