# Supplementary material for: Ingestion of Lactobacillus intestinalis and Lactobacillus reuteri causes depression- and anhedonia-like phenotypes in antibiotic-treated mice via the vagus nerve
Source: J Neuroinflammation. 2020 Aug 15;17:241. doi: 10.1186/s12974-020-01916-z (PMC7429467; doi:10.1186/s12974-020-01916-z)
Supplement: Supplementary file 1 — Additional file 1: Figure S1. Altered composition in the gut microbiota at the phylum level. (A): The relative abundances of phylum in fecal samples of the four groups 24 hrs after the final FMT. (B): Verrucomicrobi (two-way ANOVA, antibiotic: F1,24 = 6.769, P = 0.016, FMT: F1,24 = 0.128, P = 0.724, interaction: F1,24 = 1.053, P = 0.315). Data are shown as mean ± S.E.M. (n = 7). *P < 0.05. FMT: fecal microbiota transplantation. NS: not significant. W + FMT-C: water + FMT from control (no CSDS) mice. W + FMT-S: water + FMT from CSDS susceptible mice. A + FMT-S: antibiotic + FMT from CSDS susceptible mice. A + FMT-C: antibiotic + FMT from control (no CSDS) mice. Figure S2. Altered composition in the gut microbiota at the genus level. (A): The relative abundances of genus in fecal samples of the four groups 24 hrs after the final FMT. (B): Akkermansia (two-way ANOVA, antibiotic: F1,24 = 6.721, P = 0.016, FMT: F1,24 =0.107, P = 0.746, interaction: F1,24 = 0.963, P = 0.336). (C): Alistipes (two-way ANOVA, antibiotic: F1,24 = 11.641, P = 0.002, FMT: F1,24 = 9.142, P = 0.006, interaction: F1,24 = 3.879, P = 0.061). (D): Candidatus Arthromitus (two-way ANOVA, antibiotic: F1,24 = 1.064, P =0.313, FMT: F1,24 = 5.899, P = 0.023, interaction: F1,24 = 1.356, P = 0.256). (E): Parabacteroides (two-way ANOVA, antibiotic: F1,24 = 0.665, P =0.423, FMT: F1,24 = 9.407, P = 0.005, interaction: F1,24 = 3.961, P = 0.058). Data are shown as mean ± S.E.M. (n = 7). *P< 0.05, **P < 0.01. FMT: fecal microbiota transplantation. NS: not significant. W + FMT-C: water + FMT from control (no CSDS) mice. W + FMT-S: water + FMT from CSDS susceptible mice. A + FMT-S: antibiotic + FMT from CSDS susceptible mice. A + FMT-C: antibiotic + FMT from control (no CSDS) mice. Figure S3. Levels of short-chain fatty acids in fecal samples and correlation with microbiota. (A): Acetic acid (two-way ANOVA, antibiotics: F1,24 = 0.170, P =0.684, FMT: F1,24 =1.028, P =0.321, interaction: F1,24 =0.170, P =0.683) among [file 12974_2020_1916_MOESM1_ESM.docx]

**Supplemental information**

**Ingestion of *Lactobacillus intestinalis* and *Lactobacillus reuteri* causes depression-like and anhedonia-like phenotypes in antibiotic-treated mice via the vagus nerve**

Siming Wang, Tamaki Ishima, Jiancheng Zhang, Youge Qu, Lijia Chang, Yaoyu Pu,

Yuko Fujita, Yunfei Tan, Xingming Wang, and Kenji Hashimoto

Division of Clinical Neuroscience, Chiba University Center for Forensic Mental Health, Chiba 260-8670, Japan

Correspondence: Prof. Kenji Hashimoto, Division of Clinical Neuroscience, Chiba University Center for Forensic Mental Health, Chiba 260-8670, Japan

Tel: +81-43-226-2517; Fax: +81-43-226-2561 (e-mail: [hashimoto@faculty.chiba-u.jp](mailto:hashimoto@faculty.chiba-u.jp))


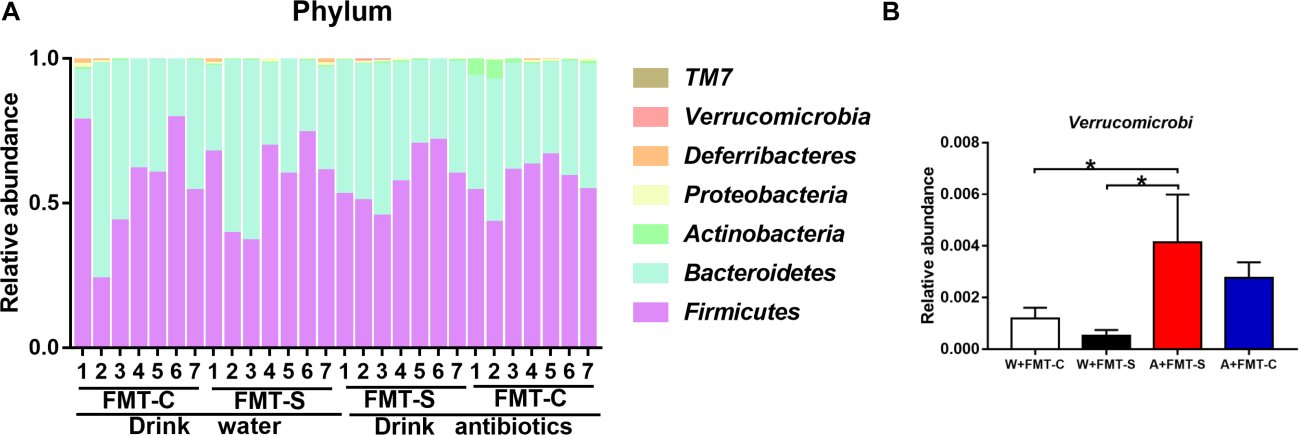


**Figure S1. Altered composition in the** **gut microbiota at the phylum level**

(A): The relative abundances of phylum in fecal samples of the four groups 24 hrs after the final FMT. (B): *Verrucomicrobi* (two-way ANOVA, antibiotic: F_1,24_ = 6.769, P = 0.016, FMT: F_1,24_ = 0.128, P = 0.724, interaction: F_1,24_ = 1.053, P = 0.315). Data are shown as mean ± S.E.M. (n = 7). *P < 0.05. FMT: fecal microbiota transplantation. NS: not significant. W + FMT-C: water + FMT from control (no CSDS) mice. W + FMT-S: water + FMT from CSDS susceptible mice. A + FMT-S: antibiotic + FMT from CSDS susceptible mice. A + FMT-C: antibiotic + FMT from control (no CSDS) mice.


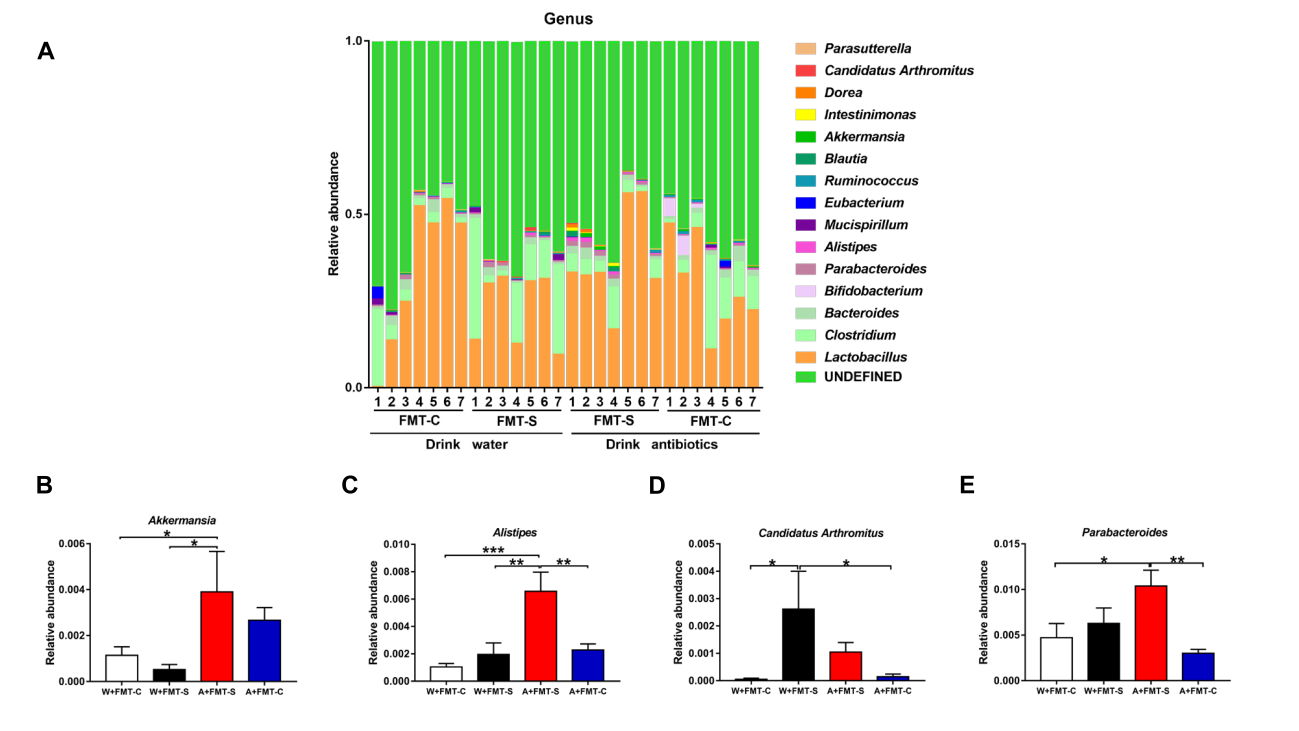


**Figure S2. Altered composition in the gut microbiota at the genus level**

(A): The relative abundances of genus in fecal samples of the four groups 24 hrs after the final FMT. (B): *Akkermansia* (two-way ANOVA, antibiotic: F_1,24_ = 6.721, P = 0.016, FMT: F_1,24_ =0.107, P = 0.746, interaction: F_1,24_ = 0.963, P = 0.336). (C): *Alistipes* (two-way ANOVA, antibiotic: F_1,24_ = 11.641, P = 0.002, FMT: F_1,24_ = 9.142, P = 0.006, interaction: F_1,24_ = 3.879, P = 0.061). (D): *Candidatus Arthromitus* (two-way ANOVA, antibiotic: F_1,24_ = 1.064, P =0.313, FMT: F_1,24_ = 5.899, P = 0.023, interaction: F_1,24_ = 1.356, P = 0.256). (E): *Parabacteroides* (two-way ANOVA, antibiotic: F_1,24_ = 0.665, P =0.423, FMT: F_1,24_ = 9.407, P = 0.005, interaction: F_1,24_ = 3.961, P = 0.058). Data are shown as mean ± S.E.M. (n = 7). *P< 0.05, **P < 0.01. FMT: fecal microbiota transplantation. NS: not significant. W + FMT-C: water + FMT from control (no CSDS) mice. W + FMT-S: water + FMT from CSDS susceptible mice. A + FMT-S: antibiotic + FMT from CSDS susceptible mice. A + FMT-C: antibiotic + FMT from control (no CSDS) mice.


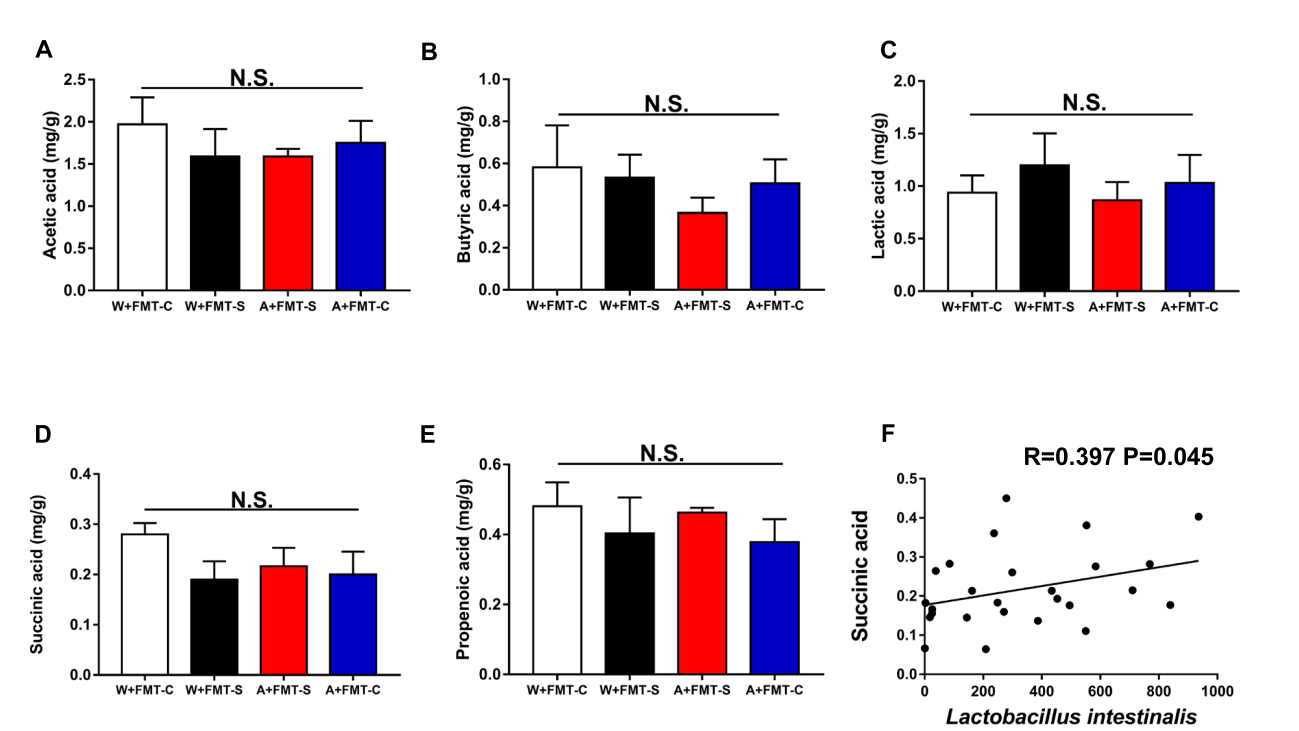


**Figure S3. Levels of short-chain fatty acids in fecal samples and correlation with microbiota**

(A): Acetic acid (two-way ANOVA, antibiotics: F_1,24_ = 0.170, P =0.684, FMT: F_1,24_ =1.028, P =0.321, interaction: F_1,24_ =0.170, P =0.683) among the four groups. (B): Butyric acid (two-way ANOVA, antibiotics: F_1,23_ = 0.831, P =0.372, FMT: F_1,23_ =0.497, P =0.488, interaction: F_1,23_=0.122, P =0.730) among the four groups.

(C): Lactic acid (two-way ANOVA, antibiotics: F_1,23_ = 0.248, P =0.623, FMT: F_1,23_ =0.038, P =0.847, interaction: F_1,23_=0.782, P =0.386) among the four groups.

(D): Succinic acid (two-way ANOVA, antibiotics: F_1,23_ = 0.511, P =0.482, FMT: F_1,23_ =0.970, P =0.355, interaction: F_1,23_=2.053, P =0.165) among the four groups.

(E): Propionic acid (two-way ANOVA, antibiotics: F_1,24_ = 0.095, P =0.761, FMT: F_1,24_ =0.003, P =0.959, interaction: F_1,24_ =1.325, P =0.261) among the four groups. (F): There is a positive correlation (r = 0.397, P = 0.045) between succinic acid and *L. intestinalis* in fecal samples. The data are shown as mean ± S.E.M. (n = 7). FMT: fecal microbiota transplantation. NS: not significant.
